# Trading in cooperativity for specificity to maintain uracil-free DNA

**DOI:** 10.1038/srep24219

**Published:** 2016-04-11

**Authors:** Judit E. Szabó, Enikő Takács, Gábor Merényi, Beáta G. Vértessy, Judit Tóth

**Affiliations:** 1 Institute of Enzymology, Research Centre for Natural sciences, Hungarian Academy of sciences, Budapest, Hungary; 2 Department of Applied Biotechnology and Food Science, Budapest University of Technology and Economics, Budapest, Hungary

## Abstract

Members of the dUTPase superfamily play an important role in the maintenance of the pyrimidine nucleotide balance and of genome integrity. dCTP deaminases and the bifunctional dCTP deaminase-dUTPases are cooperatively regulated by dTTP. However, the manifestation of allosteric behavior within the same trimeric protein architecture of dUTPases, the third member of the superfamily, has been a question of debate for decades. Therefore, we designed hybrid dUTPase trimers to access conformational states potentially mimicking the ones observed in the cooperative relatives. We studied how the interruption of different steps of the enzyme cycle affects the active site cross talk. We found that subunits work independently in dUTPase. The experimental results combined with a comparative structural analysis of dUTPase superfamily enzymes revealed that subtile structural differences within the allosteric loop and the central channel in these enzymes give rise to their dramatically different cooperative behavior. We demonstrate that the lack of allosteric regulation in dUTPase is related to the functional adaptation to more efficient dUTP hydrolysis which is advantageous in uracil-DNA prevention.

Allosteric characteristics of protein-ligand interactions present a research field with great traditions but also with key current interest^1^,^2^. Recent developments in the understanding of the manifestation of allosteric behavior within a given protein architecture lead to a shift from the classical view to a more subtle dynamic view of allostery. According to the classical view, allostery is based on a series of distinct structural changes leading to functionally different conformers of a given protein. However, several examples demonstrated that the allosteric behavior does not necessarily require switches between direct conformers^3^ but can be explained by alterations of side chain^4^ or main chain dynamics^5^ or by a shift in the distribution of preexisting protein conformations^6^. In the present study, we set out to investigate the manifestation of allostery within the superfamily of dUTPases. The superfamily comprises the dUTPase, dCTP deaminase (DCD) and the bifunctional dCTP deaminase-dUTPase (DCD-DUT) enzymes that produce dUMP, the obligatory *de novo* dTTP synthesis precursor from either dUTP or dCTP, respectively (Fig. 1).

The removal of dUTP from the cellular dNTP pool is also a vital function of dUTPases. These enzymes thus play an important role in the maintenance of the pyrimidine nucleotide balance and genome integrity^7^,^8^,^9^,^10^,^11^,^12^. dUTPase and DCD(-DUT) share a common homotrimeric structural core^13^,^14^,^15^,^16^ (Fig. 2a). The three subunits form a central channel and three equivalent active sites at the intersubunit clefts (Fig. 2a). This intricate quaternary structure intuitively suggests the possibility for allosteric control within the enzyme. In effect, the allosteric communication between the active sites of DCD family enzymes has been investigated in several species^15^,^17^,^18^,^19^ and was found to operate through the central channel^15^,^19^. The cooperative conformational change in these enzymes occurs in a loop (referred to as allosteric loop from now on) located at the interface of the nucleotide binding site and the central channel (Fig. 2b). The allosteric loop can adopt the mutually exclusive active and inactive conformations. The conformational change of one loop facilitates the same conformational change in the other two loops of the trimer due to steric hindrance within the central channel^15^,^19^. This mechanism thus conforms to the classical view of allostery.

dUTPases, the other family that belongs to the dUTPase superfamily, display an even more intricate interaction pattern between their subunits than DCDs do. The C-terminal arm of dUTPases in almost all cases reaches across the trimer to the remote active site and therefore, all three subunits provide conserved residues to each active site^16^. This structure inspired the proposition that allosteric communication between the active sites of dUTPases should also exist^20^,^21^,^22^. Crystallographic observations in the human dUTPase suggested that some allosteric effect must help the release of the dUMP product^20^. Another study investigating the nature of the central channel of dUTPases found considerable difference in hydropathy between eukaryotic and prokaryotic dUTPases^21^. It was proposed that allostery can emerge through the hydrophilic central channel in eukaryotic dUTPases. A later NMR study suggested that the *Drosophila* enzyme exhibits cooperativity in both substrate and product binding based on signal intensity titrations well above the K_d_ of the respective complexes^22^. In the EIAV (Equine Infections Anemia Virus) enzyme, a Trp at the central channel senses the nucleotide-bound states of the active site^23^ corroborating the potential of active site communication through the central channel. On the other hand, detailed kinetic analyses of dUTPases from various species (human^24^, *E. coli*^25^, EIAV^23^, *Plasmodium falciparum*^26^) failed to directly detect any cooperative behavior in the enzymatic mechanism.

The cooperativity in DCD is best observed when dTTP, its feedback inhibitor binds to the active site. As the accommodation of the additional metal group on the thymine ring is possible only in the inactive conformation^15^,^19^, the shift in the equilibrium between the active and inactive conformational states and the cooperative behavior is more pronounced in the presence of dTTP^15^,^19^,^27^. The substrate binding pocket of dUTPases, however, does not accommodate other bases than uracil with considerable affinity^20^,^25^,^28^. This property of dUTPases may make it difficult to recognize any allosteric behavior. The inherent allosteric potential in proteins may only appear by mutations that shift the distribution of the various conformational states^6^,^29^. Therefore, we designed mutations to access inactive conformational states in dUTPase potentially mimicking the ones observed in DCD and DCD-DUT. To this end, we created covalently linked human dUTPase pseudoheterotrimers (called hybrids henceforth) in which the active sites could be turned off selectively (Fig. 2c). We studied how the interruption of different steps of the enzyme cycle in one active site of the hybrid affects the activity of the non-mutated active sites using various enzymatic and structural biology approaches. Our experimental results combined with the comparative analysis of the structural features of dUTPase superfamily enzymes reveals an intriguing trade-off between regulation and efficiency, two ways of functional adaptation to distinct metabolic functions.

## Results

### Establishment and enzyme activity of asymmetric dUTPase hybrids

Mutations introduced to a homooligomeric protein appear in each subunit as the oligomer is assembled from identical monomers (Fig. 3a). To generate hybrid enzymes of the human dUTPase (hDUT), we therefore needed to create a covalently linked pseudohomotrimer (termed WWW) in which each subunit could be exchanged to selectively contain the desired mutation (Fig. 3b). The WWW construct was assembled from the previously described sensor-bearing hDUT^F158W^ human dUTPase^24^,^30^ monomers (W) connected with flexible peptide linkers^31^ (see Online Methods and Supplementary Table 1). We previously used the F158W substitution to follow the enzymatic reaction steps using the intrinsic fluorescent signal of tryptophan 158^24^,^32^,^33^. In this position, a conserved aromatic residue overlaps with the uracil ring of the substrate in every dUTPase^33^. The electrostatic interaction between the tryptophan and uracil modifies the fluorescence properties of the tryptophan residue in a conformation-sensitive manner allowing the monitoring of the enzymatic cycle. The WWW enzyme could be expressed and purified (Fig. 3c) with similarly high yield as the homotrimer. We introduced each further mutation into the WWW scaffold by exchanging one or more of the W cassettes.

The sequence of the linker (ASGAGGSEGGGSEGGTSGATG/SL/Q) was borrowed from a study in which it was used for the same purpose: genetic manipulation of individual subunits. The visible N- and C-terminal amino acids of hDUT can be found 28.62 Å apart in the 2HQU structure. The 22 amino acid linker spans 77 Å when maximally streched. The flexible N-terminal 23 amino acids add another 80.5 Å streched length to the linker. Therefore, we anticipated that this linker does not restrict the wild-type dynamics of the hDUT structure. To test whether the linkers influence the enzymatic properties of the WWW enzyme, we applied transient kinetic analysis using the fluorescent signal of W158. The kinetic parameters of substrate binding and hydrolysis as well as the characteristic fluorescence changes during the course of the reaction remained similar in WWW to that measured in hDUT^F158W^ (Supplementary Fig. 1, Table 1). We also performed limited tryptic digestion of the covalent enzyme to cleave the linkages between the subunits. The cleaved enzyme displayed basically identical steady-state and transient kinetic properties to that of the covalently linked one (Supplementary Fig. 2). We concluded that the linkers do not influence the enzymatic mechanism and thus the covalent enzyme is suitable for further investigations.

Four different hybrid enzymes were subsequently created to investigate the possible allosteric effects of conformational changes upon substrate binding, hydrolysis or product release (Fig. 4a).

To investigate the allosteric effect of a possible global conformational change upon substrate binding, we created an active site that is unable to accommodate the uracil ring (Supplementary Fig. 3a) and is thus defective in substrate binding and any conformational change coupled to it. Control experiments with the homotrimeric form of the A98F mutant (hDUT^F158W, A98F^) confirmed that it could not bind the substrate and was entirely inactive (Supplementary Fig. 3b–f). We introduced this mutation to the covalent construct to obtain WWF.

The following constructs were designed to investigate whether conformational changes occurring upon hydrolysis or product release are necessary to be transmitted to neighboring active sites for the global activity of the trimer.

In the WWN and WNN constructs, the conserved catalytic Asp from the third conserved motif was changed to Asn (Supplementary Fig. 3g) in one or two active sites, respectively. This Asp/Asn substitution has been described to reduce the catalytic activity close to zero while the substrate binding properties do not change^34^. We also tested the enzymatic properties of hDUT^D102N,F158W^ and found that the catalytic activity decreased below the detectable level (<0.002 s^−1^) both under steady-state and single turnover conditions while the substrate binding properties remained unaffected compared to hDUT^F158W^ (Supplementary Fig. 3h).

In the WWS enzyme, we removed the C-terminal P-loop-like motif of the last subunit of the pseudoheterotrimer. This conserved motif interacts with the γ-phosphate of dUTP and stacks over the uracil ring to orient the catalytic apparatus and stabilize the transition state^30^,^32^,^33^,^35^. The P-loop-like motif is only present in dUTPases and is missing from DCDs or DCD-DUTs. Its removal results in major decrease (~720 fold) of the catalytic constant and minor (~3 fold) increase in the dissociation constant of the enzyme-substrate complex^30^.

### dUTPase active sites work independently from each other

Potential allosteric interactions within the hybrids WWN, WNN, WWS and WWF were analyzed using the combination of steady state and transient kinetic measurements. All constructs proved to be active indicating that the arrest of the enzyme cycle in a given active site does not compromise the enzymatic turnover in the others. Steady-state activity titrations of all hybrid enzymes exhibited Michaelis-Menten kinetics (Fig. 4b). The maximal initial velocities (V_max_) decreased proportionally with the number of inactivated sites, i.e. WWN, WWF and WWS displayed about 2/3, while WNN displayed approximately 1/3 activity compared to WWW (Fig. 4c, Table 1). This indicates that the activity per working subunit is unaltered in the hybrid enzymes. The Michaelis constants did not change considerably compared to the WT (Table 1). Single turnover stopped-flow measurements showed that the kinetic mechanism of the asymmetric hybrids is identical with that of the control WWW (Supplementary Fig. 4). The k_obs_ for substrate binding and the single turnover rate constants (k_STO_) remained unaltered (Table 1, Fig. 4c). This implies that the observed decrease in the steady-state activity is only due to the decreased active site concentration.

The kinetics of substrate binding to the hybrid enzymes was also investigated under pseudo first order conditions. A large part of the time courses got lost in the dead-time of the instrument which hindered the determination of the rate constants. The total signal change, however, could be used to determine the dissociation constants (K_d_) of the enzyme-dUTP complexes (Fig. 4d). The obtained K_d_-s were similar to that of the WWW-dUTP complex (Table 1).

In summary, a global active to inactive conformational transition observable in DCDs could not be identified in dUTPase even in conditions potentially mimicking the asymmetry in a partially dTTP-saturated DCD enzyme (Fig. 2c). On the contrary, Fig. 4c clearly indicates that the active sites turn over independently from each other. In case of a cooperative transition to a global inactive state we would expect inactivity following the first turnover or non-proportional activity decrease in the hybrid enzymes containing one or two defective active sites.

### The conformational flexibility of human dUTPase is restricted by Mg^2+^ binding to the central channel

The conformational changes resulting in the observed cooperative behavior is transmitted through the central channel in DCD family enzymes. We therefore investigated the structural features of the channel possibly responsible for the lack of conformational transmission in dUTPase. The site of cooperative conformational change of DCD(-DUT) enzymes corresponds to one of the two suggested Mg^2+^ binding sites in the human dUTPase^20^,^36^ (Asp95, Fig. 5a). In contrast, no metal binding to DCD(-DUT) enzymes has been reported.

To evaluate the role of Mg^2+^ binding to the central channel of hDUT^F158W^, we conducted in solution structural investigations in the presence and absence of Mg^2+^. The near-UV CD spectra showed considerable changes upon the addition of Mg^2+^ to the apo hDUT^F158W^ indicating that the metal ion binds to the enzyme and modifies its structure (Fig. 5b). The largest signal change was observable at 285 nm probably yielded by the rearrangement of Tyr residues (Fig. 5a). The far-UV CD spectra, on the other hand, showed only minor changes upon Mg^2+^ addition (Fig. 5b inset) implying that metal binding may not induce major changes in the secondary structure. The WWW enzyme showed similar spectral changes to hDUT^F158W^ upon the addition of Mg^2+^ (Supplementary Fig. 5a,b).

The thermal denaturation of hDUT^F158W^ could be described with a two-state equilibrium model indicating that the heat-induced unfolding of the trimer happens in one step, without a significantly populated intermediate state of dissociated and folded monomers^37^ (Fig. 5c). In the absence of Mg^2+^, we observed a slight but reproducible decrease in the melting temperature (T_m_) (Fig. 5c) suggesting that the stability of the enzyme is slightly decreased in the absence of Mg^2+^ similarly to what was found in the *D. melanogaster* dUTPase^38^. The covalent WWW enzyme produced a more complicated melting curve (Supplementary Fig. 5c). However, the stabilization effect of Mg^2+^ could also be observed.

To test the potentially increased flexibility of hDUT^F158W^ in the absence of Mg^2+^, we performed limited trypsinolysis. hDUT^F158W^ was highly sensitive to tryptic digestion in the absence of Mg^2+^ (Fig. 5d and Supplementary Fig. 6) whereas the control *Mycobacterium tuberculosis* dUTPase (mtDUT) that does not contain Mg^2+^ binding sites in its central channel (Fig. 6f) was not (Fig. 5d and Supplementary Fig. 6). Following the expected cleavage of the flexible N- and C-termini^32^, the remaining enzyme core – which is otherwise stable for long time – disappeared within an hour (Fig. 5d). In case of the mtDUT, the enzyme core remained stable during the one our experiment. This phenomenon indicates that the quaternary structure of hDUT^F158W^ is significantly more flexible in the absence than in the presence of Mg^2+^.

### Structural comparison of dUTPase superfamily enzymes reveals trade-off in conformational flexibility and active site specificity

To understand the structural basis of the mechanistic differences within the dUTPase superfamily, we compared the central channel, the region of the allosteric loop and the nucleobase binding region in DCDs and dUTPases (Fig. 6).

The amino acids responsible for the binding of the nucleobase are located around motif 3 (Fig. 6a) at the N- and C-terminal parts of the β-hairpin accommodating the nucleoside (Fig. 6b). The N-terminal few amino acids of the β-hairpin overlap with the allosteric loop in the DCD family. We found that this region is highly diverse in size and amino acid composition within the superfamily (Fig. 6a,b).

In eukaryotic dUTPases, the loop is shorter by 2 or 3 amino acids than in DCDs resulting in a tighter uracil binding cleft. In these enzymes, mostly main chain atoms establish hydrogen bonding interactions with the nucleobase, which was proposed to contribute to their specificity for uracil^20^. The very same loop confers the Mg^2+^ binding site facing the central channel (Fig. 6c, Fig. 5a).

In retroviral dUTPases (EIAV shown as the representative), the loop is even shorter than that of the Mg^2+^ binding site-bearing eukaryotic dUTPases (Fig. 6a,b).

The allosteric loop of prokaryotic dUTPases is of the same length as that of DCDs. However, its amino acid composition is different (Fig. 6a) resulting in a tighter central channel mainly due to the LV/SM/SP/AP peptides protruding in it (Fig. 6a,b). The conformational flexibility of the channel is restricted by the hydrophobic interactions of the modified allosteric loop (Fig. 6d) or by a conserved Pro (Fig. 6e). In DCDs, the conserved Ala of the allosteric loop engages in H-bonding with the oxo group of the substrate uracil. If thymine is bound to the active site, the HVTA peptide containing this Ala moves into the channel (cf. Fig. 2b). In contrast, a conserved Asn plays the same H-bonding role in prokaryotic dUTPases but it resides in a conformationally restricted peptide (Fig. 6a,d,e).

The sequence and structural comparison with DCD enzymes reveals that the various evolutionary branches of dUTPases possess an altered or shortened allosteric loop which coincides with the conformational stabilization of the central channel. Interestingly, most of the observed alterations contribute to the specificity for dUTP at the same time. We propose that the central channel of dUTPases features increased stability at the cost of lacking the potential for mediating cooperativity as observed in DCDs. Apparently, the same structural element of the active site is responsible for substrate specificity and for the communication through the central channel.

## Discussion

**Figure 1:**
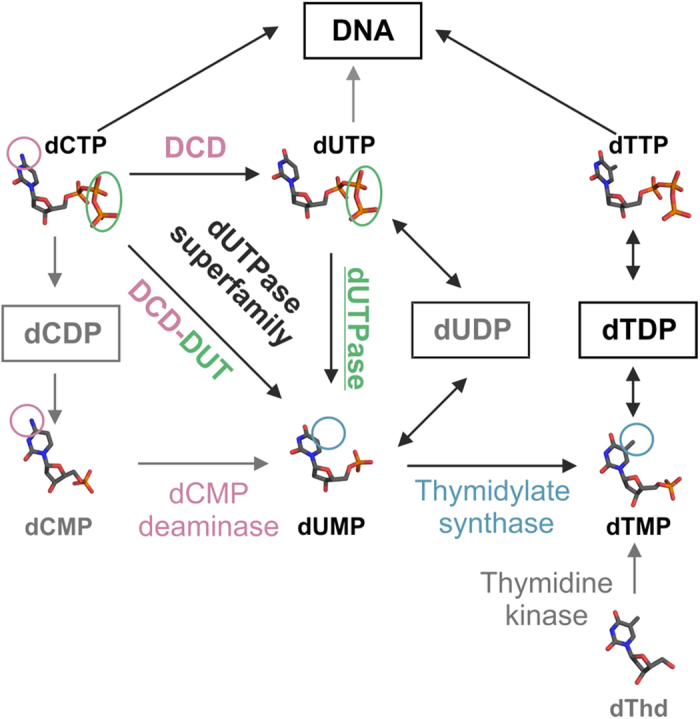
Overview of the dTTP biosynthesis pathways. The three dimensional structures of the nucleotides were extracted from pdb files (dCTP: 1XS4; dUTP: 2V9X; dUMP: 1SNF; dTMP: 1TMK; dTTP: 2QXX; dCMP: 1B5E; dThd: 2V9X) and are shown as atomic colored sticks (C: grey, O: red; N: blue; P: orange). Circled chemical groups call attention to the differences relevant to the enzymatic reactions that transform pyrimidine nucleotides into each other.

**Figure 2:**
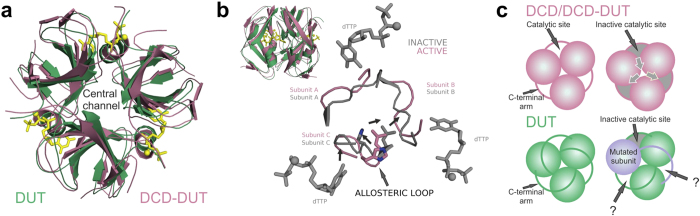
Structure and communication in the homotrimers of the dUTPase superfamily. (**a**) “Topview” of the superpositioned core structures of a dUTPase (from *Mycobacterium tuberculosis*, PDB: 2PY4) and a bifunctional DCD-DUT (also from *Mycobacterium tuberculosis*, PDB: 2QLP). The substrate analog dUPNPP (yellow sticks) in complex with Mg^2+^ ions (yellow spheres) is also shown within the dUTPase structure to highlight the active sites. (**b**) Upper left corner: “side” view of the superpositioned enzyme cores from panel (**a**). The position of the enlarged cross-section plane is indicated by the yellow sticks. Concerted conformational switch within the central channel of DCD-DUT is shown by superposing the apo enzyme in active conformation (PDB: 2QLP) with the dTTP bound enzyme in inactive conformation (PDB: 2QXX). Arrows highlight the most important conformational changes. Note, that only the inactive conformation can accommodate the methyl group of dTTP (grey sticks, Mg^2+^ ions: grey spheres). (**c**) Schematic representation of active site communication within the dUTPase superfamily. Grey color at the active sites represents enzymatic inactivity. In DCD (-DUT), arrows in the central channel indicate that the inactive conformation is spread through the central channel in a concerted way.

**Figure 3:**
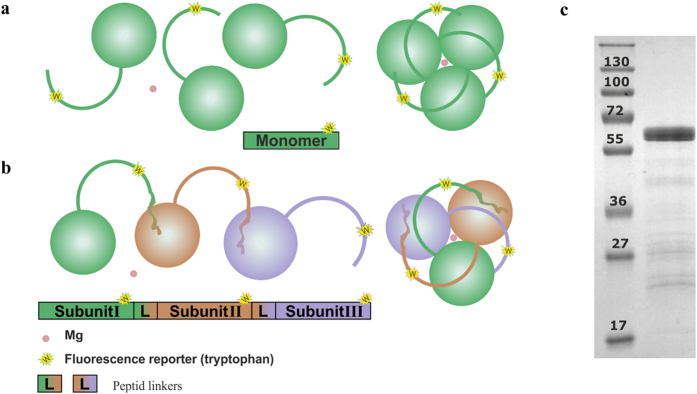
Schematic representation of the covalent trimer concept. (**a**) Schematic representation of the assembly of the hDUT^F158W^ dUTPase. Green spheres represent the sensor-bearing hDUT^F158W^ subunits. The structures reaching out from the subunits represent the swapping C-terminal arm of dUTPase. Yellow stars mark the tryptophan serving as intrinsic fluorescence signal of the enzyme conformations. The pink sphere indicates a Mg^2+^ ion. (**b**) Schematic representation of the covalent pseudotrimers. The projection of the swapping arms with color transition represents the flexible linkers connecting the C-terminus of one dUTPase protomer to the N-terminus of another one. Different colors of the protomers represent the possible heterogeneity within the trimer, i.e. every protomer can be changed independently from each other. (**c**) SDS-PAGE analysis of the purified WWW enzyme. The main band is located at the expected position which corresponds to the calculated molecular weight of 58064 Da.

**Figure 4:**
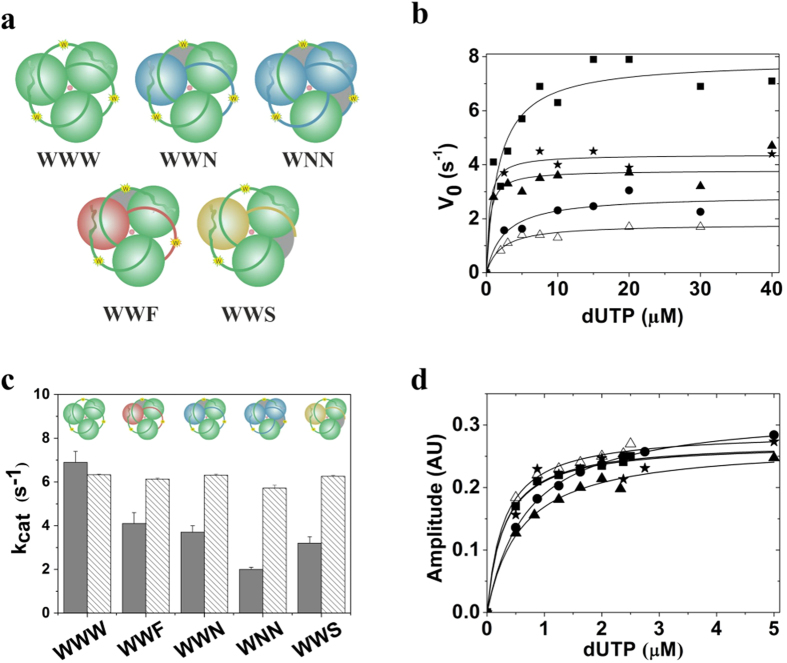
Asymmetric hybrid enzymes exhibit non-cooperative kinetics in the different reaction steps. (**a**) Schematic representation of the created hybrids (covalent heterotrimers). Blue, red and yellow spheres represent dUTPase protomers containing the D102N, A98F and T148STOP mutations, respectively. Note, that all protomers contain the F158W mutation as well, except for the T148STOP mutant. Grey areas indicate enzymatic inactivity. (**b**) Steady-state kinetics of human dUTPase constructs: WWW (solid square), WWN (solid triangles), WNN (open triangles), WWF (solid stars), WWS (solid circle). Smooth lines through the data are hyperbolic fits yielding V_max_ = 7.9 ± 0.5 s^−1^ for WWW, V_max_ = 4.4 ± 0.2 s^−1^ for WWF, V_max_ = 3.8 ± 0.2 s^−1^ for WWN, V_max_ = 1.8 ± 0.1 s^−1^ for WNN, V_max_ = 2.9 ± 0.3 s^−1^ for WWS. K_M_ values are listed in Table 1. (**c**) Comparison of the catalytic constants (striped bar) and apparent catalytic constants (grey bar) for determined by single turnover (transient kinetics) and steady-state experiments, respectively. See also Table 1 for the data. (**d**) Fluorescence intensity titrations upon dUTP binding to the various dUTPase constructs measured by stopped-flow (the symbol code is identical to that in panel (**b**). Smooth lines through data are hyperbolic fits yielding K_d_ values summarized in Table 1.

**Figure 5:**
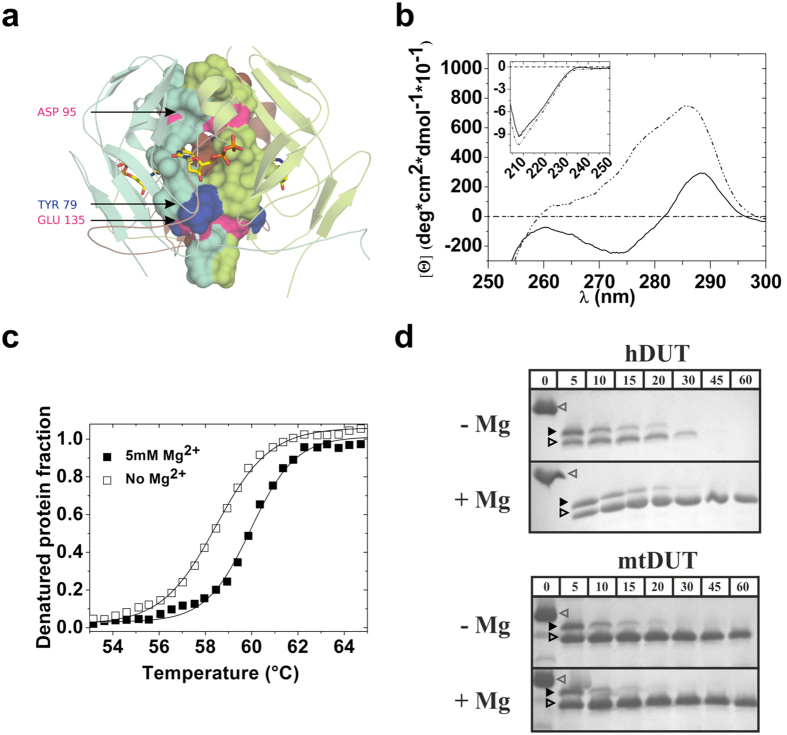
Mg^2+^ binding to the central channel reduces the flexibility of the dUTPase trimer. (**a**) Predicted Mg^2+^ binding sites (pink) within the central channel of human dUTPase (PDB: 1Q5H, colored by subunits). The residues constituting the channel wall are shown as surface while the rest of the molecule is shown as cartoon representation. The Tyr residues possibly responsible for the change in the near UV spectra upon Mg^2+^ binding are shown in blue. Active sites are highlighted by the bound dUDP (shown as sticks with atomic coloring). (**b**) Near UV and Far UV (inset) spectra of hDUT^F158W^ in the presence (dash-dot-dot) and in the absence (solid line) of 5 mM MgCl_2_. The spectrum of the buffer is marked by dash-dot line. (**c**) Thermal unfolding of hDUT^F158W^ in the presence and in the absence of 5 mM Mg^2+^. Smooth lines through the data are Boltzmann fits (Equation (4)). The melting temperatures (transition midpoints) are Tm = 59.8 ± 0.2 °C in the presence of MgCl_2_ and Tm = 58.4 ± 0.2 °C in the absence of MgCl_2_ (n = 3). Errors represent SD. (**d**) Limited trypsinolysis of hDUT^F158W^ and mtDUT^H145W^ performed in the presence and in the absence of 5 mM MgCl_2_. The open gray arrow head, the black arrow head and the open black arrow head indicate the intact, the N-terminal cleaved and the N- and C-terminal cleaved enzymes, respectively. The densitometric analysis of the relative amount of the core enzyme (intact enzyme + N-terminal cleaved enzyme + N- and C-terminal cleaved enzyme) is shown in Supplementary Fig. 5d.

Allosteric enzyme regulation is one of the general means of controlling biochemical processes. The appropriate concentration and balance of dNTPs for DNA synthesis and repair is commonly regulated by both homotrop and heterotop allosteric mechanisms^39^,^40^. Feedback inhibition by dTTP in two of the three dUTPase superfamilies, DCDs and the bifunctional DCD-DUTs (Fig. 1), seems to be important to maintain the appropriate dCTP/dTTP ratio^15^,^19^. The feedback inhibitor dTTP binds to the active sites which communicate with each other within the homotrimer resulting in an all-or-none inhibition pattern. This is a more complicated and more efficient inhibition mechanism than simple competitive inhibition due to the fact that 1 dTTP molecule elicits the complete inhibition of 3 active sites. Interestingly, the activity of the non-homologous dCMP deaminases is also modified by dCTP and dTTP with intricate regulation pattern involving cooperativity^41^,^42^,^43^. Probably, this kind of regulation is important in maintaining the correct dCTP/dTTP ratio^44^.

We attempted to detect allosteric behavior in dUTPases by engineering hybrid enzymes to restrict putative allosteric transmission between active sites at various stages of the enzymatic cycle. Interestingly, however, the enforced asymmetry in the dUTPase trimer did not elicit any instance of cooperative behavior despite the fact that dUTPases have the most intertwined trimeric structure of all within the dUTPase superfamily. We determined that i) the active sites work independently from each other; ii) Mg^2+^ binding in the central channel reduces the flexibility and increases the thermal stability of the quaternary structure; iii) the allosteric loop that connects the active site to the central channel is conformationally restricted in dUTPases compared to DCD family enzymes. This phenomenon is interrelated with structural solutions for increased dUTP specificity in every case.

**Figure 6:**
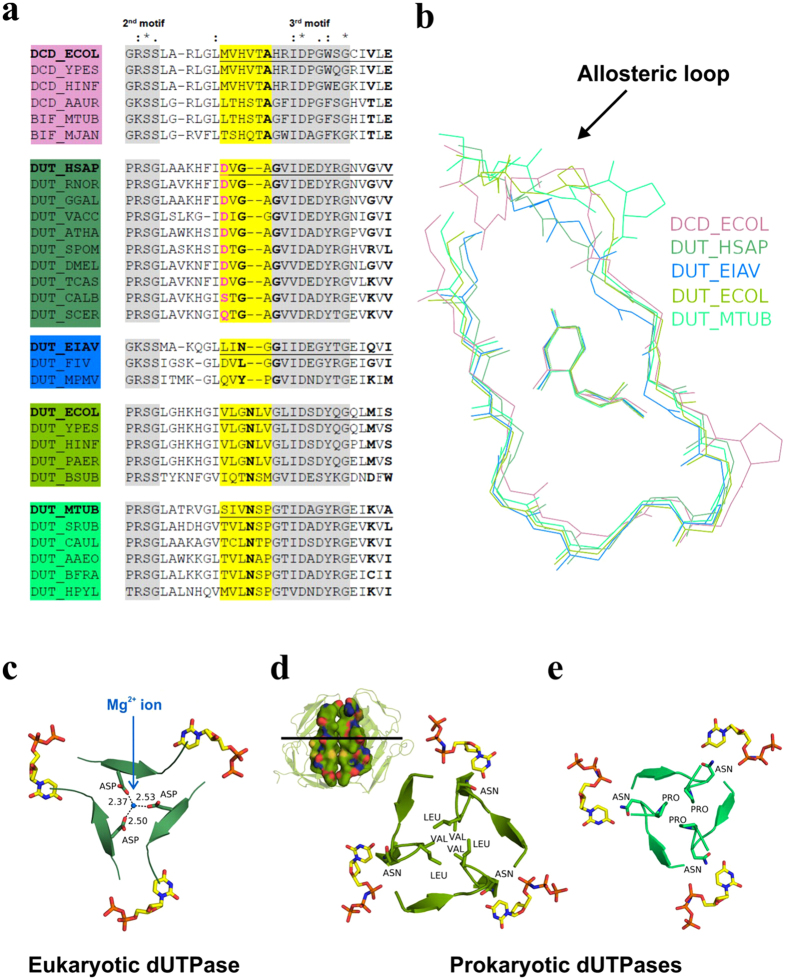
Stabilization of the central channel hinders conformational coupling with the active sites in dUTPases. (**a**) Sequence comparison of the allosteric loop region within the dUTPase superfamily. The alignment was created by clustalW with minimal manual editing. Allosteric loop, yellow highlight; the uracil binding cleft, underlined; conserved motifs, grey highlight; uracil ring coordination, bold. Amino acid conservation is distinguished by: identity (*), strong similarity (:) and weak similarity (.). (**b**) Superposition of the 3D structures of representative dUTPase superfamily enzymes from panel (**a**) (PDB: *E. coli* DCD – 1XS1, hDUT – 2HQU, EIAV DUT – 1DUC, *E. coli* DUT – 1RN8, MTB DUT – 3HZA). The superposition was performed by the alignment of the bound nucleotides using PyMol. Only the main chain atoms of the proteins and the nucleoside part of the ligands are shown for clarity. The color code refers to the proteins in panel (**a**). Note the structural variance in the allosteric loop. (**c–e**) Cross section of the central channel of hDUT (1Q5H), *E. coli* DUT and MTB DUT at the level of the uracil binding pocket. The side chains within the central channel and the bound ligands are shown as sticks with atomic coloring. The Mg^2+^ ion is represented as blue non-bound sphere. In panel **(d)** the longitudinal view of the threefold trimer interface (two subunits are shown) of the *E. coli* DUT is also shown using surface representation with atomic coloring to highlight the hydrophobic character of the central channel.

It is common in enzyme evolution that mutations in flexible active site loops are responsible for altered substrate specificity^45^. Since active sites are often located at intersubunit/interdomain clefts, these flexible loops have a potential to mediate allosteric communication between the active sites. In our case, it seems that the specialization for a single substrate results in the loss of allosteric communication. The shorter/less flexible allosteric loop compared to that of DCDs and DCD-DUTs is related to increased dUTP specificity (Fig. 6). Another dUTPase “invention” is the C-terminal P-loop-like motif that discriminates against dUDP and makes dUTP hydrolysis more efficient by several orders of magnitude than DCD-DUTs^30^. The gain of specificity together with the enhancement of the catalytic power represent features that make dUTPases significantly more powerful in dUTP breakdown as compared to DCD-DUTs. This advance may be necessary to avoid the appearance of the non-canonical uracil base in DNA that induces the activation of DNA repair mechanisms upon non-physiological uracil accumulation leading to severe to fatal consequences for the cell^46^,^47^,^48^. The cooperative allosteric behavior in DCDs and DCD-DUTs, on the other hand, make these enzymes suitable for the regulation of the nucleotide pool. We propose that the trade-off between cooperativity and specificity in the dUTPase superfamily represents instances of adaptation to the distinct roles of dUMP production for dTTP synthesis and dUTP elimination for uracil-DNA avoidance, respectively.

## Methods

### Reagents

Molecular biology products were from New England Biolabs (US) and Fermentas (Canada), electrophoresis and chromatography reagents were from Bio-Rad (US) and Qiagen (Netherland). Phenol red was from Merck (Germany). dUMP, dUDP and α,β-imido-dUTP (dUPNPP) was from Jena Bioscience (Germany), dUTP and other chemicals were from Sigma–Aldrich (US).

### Cloning and mutagenesis

Site-directed mutagenesis was performed by the QuikChange method (Stratagene) and was verified by sequencing. The enzyme conferring a tryptophan sensor in the active site (hDUT^F158W^) was used as wild-type^24^,^32^,^33^. The following mutants were created within this construct: Asp102 to Asn (hDUT^F158W,D102N^) and Ala98 to Phe (hDUT^F158W,A98F^). Mutagen forward and reverse primers are presented in Supplementary Table 1. The covalent wild type enzyme (WWW) was created by genetic engineering. hDUT^F158W^ was amplified with primers encoding linkers and various restriction sites (Supplementary Table 1) to create the three subunits of WWW. The subunits were cloned to pET45b plasmid as individual restriction cassettes. Covalent pseudoheterotrimers (hybrids) were then created by changing one or two of the wild type cassettes in WWW to mutant one(s).

### Protein expression and purification

Expression and purification of noncovalent human dUTPase proteins were done as described previously in Varga *et al.* FEBS Letters^32^. Covalent dUTPase pseudotrimers were expressed and purified similarly, except that BL21 Rosetta (pLysS) cells (Novagen) were used instead of BL21. The expression and purification of the His-tagged *Mycobacterium tuberculosis* dUTPase (mtDUT) was done based on Varga *et al.* BBRC^49^. The protein concentration was measured using the Bradford method (Bio-Rad Protein Assay) and by UV absorbance. Extinction coefficients were calculated based on the amino acid sequence using the ProtParam tool (http://web.expasy.org/protparam/). Extinction coefficients for the proteins were: λ_280_ = 16055 M^−1^cm^−1^ for the hDUT^F158W^, hDUT^F158W,D102N^ and hDUT^F158W, A98F^; λ_280_ = 48290 M^−1^ cm^−1^ for the WWW, WWF, WWN and WNN and λ_280_ = 42790 M^−1^ cm^−1^ for the WWS construct. Protein concentration is given in monomer/subunit concentration in every case. All measurements were carried out in a buffer comprising 20 mM HEPES pH 7.5, 100 mM NaCl, 2 mM MgCl_2_ and 2 mM ß-mercaptoethanol (“assay buffer”) if not stated otherwise.

Steady-state colorimetric dUTPase assay was performed as described in Varga *et al.* BBRC^49^. This phenol red indicator assay was used to detect the protons released in the dUTPase reaction. 0.01–1 μM protein was used for the dUTPase assay in a buffer containing 1 mM HEPES pH 7.5, 100 mM KCl, 40 μM phenol red and 5 mM MgCl_2_. A Specord 200 spectrophotometer (Analytic Jena, Germany) and 10 mm path length thermostatted cuvettes were used at 20 ^o^C and absorbance was recorded at 559 nm. The initial velocity was determined from the first 10% of the progress curves. Initial velocities were plotted against substrate concentration and the results were fitted with the Michaelis–Menten equation.

### Fluorescence measurements

Fluorescence spectra and intensity titrations were recorded on a Jobin Yvon Spex Fluoromax-3 spectrofluorometer in the assay buffer at 20 ^o^C. Trp fluorescence was excited at 297 nm, emission spectra were recorded between 320–400 nm while the fluorescence intensity titrations were detected at 345 nm. Additional fluorescence or inner filter effect imposed on the measured intensities during the titration experiments were corrected by subtracting the corresponding buffer spectra. Titration data were fitted with the equation describing 1:1 stoichiometry for the dissociation equilibrium assuming no cooperativity:


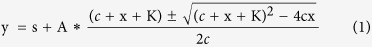


where *x* is the ligand concentration and *y* is the fluorescence intensity, *s* = *y* at *x* = 0, *A* is the amplitude of the fluorescence intensity change, *c* is the enzyme concentration and *K* is the dissociation constant of the ligand complex.

### Thermofluor assay

Thermal shift assays were carried out on an Mx3000P^®^ QPCR System (Agilent Technologies Company). Thermal shift reactions were performed in a 96-well thin-wall microplate in a total volume of 25 μl containing 500× diluted Sypro® Orange dye. Samples were heated from 25.0 to 80.0 ^o^C. The speed of heating was 1 °C/minute. The protein concentration of hDUT^F158W^ was 0.8 mg/ml in the measurements, while the WWW enzyme was used at 2 mg/ml concentration. To compensate for the difference in the ionic strength between the samples with and without MgCl_2_, NaCl was added according to Equation 2:


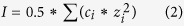


where *I* is the ionic strength, *c*_*i*_ is the concentration, *z*_*i*_ is the charge of the particular ion and *i* is the index of summation.

The raw data of the heat-induced unfolding monitored by fluorescence emission were converted to the apparent fraction of native protein *F*_*N*_, according to Equation (3):


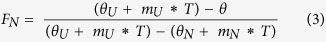


where *θ* is the observed spectroscopic signal at temperature *T*, *θ*_*N*_ and *θ*_*U*_ are the intercepts and *m*_*N*_ and *m*_*U*_ are the slopes of the pre- and post-transitional base lines of the raw data, respectively. The *F*_*N*_ vs. *T* plot was converted to the *F*_*U*_ vs. *T* plot by using the *F*_*N*_ + *F*_*U*_ = 1 equation, where *F*_*U*_ is the fraction of unfolded protein. The *F*_*U*_ vs. *T* plot was then fitted with the Boltzmann (Equations (4)) the midpoint of the transition


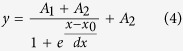


where *A1* and *A2* are the pre- and post-transitional base lines and *x*_*0*_ is the transition midpoint.

### Circular dichroism measurements

CD spectra recording was carried out in a JASCO 720 spectropolarimeter at 20 ^o^C using a quartz cuvette with 1 mm (far UV) or 10 mm (near UV) path length. Far UV and near UV spectra were recorded at 200–250 nm or 250–300 nm, respectively. All protein containing spectra were corrected by subtracting the corresponding buffer spectra.

### Fast kinetics experiments

Fluorescence stopped-flow measurements were carried out at 20 ^o^C using an SX-20 stopped-flow apparatus (Applied Photophysics, UK) as described previously^24^. Equal volumes (50 μl) of dUTPase enzyme and dUTP solutions were mixed and 8 traces were recorded and averaged for each time course. Under single turnover conditions, a triple exponential equation was fitted to the averaged traces to determine the catalytic constants based on Tóth *et al.* JBC^24^. For the determination of binding rate constants, the ligand titration was performed under pseudo-first order conditions. The observed rate constants for the two binding steps described previously (collision complex formation and isomerisation^24^) were determined by fitting double exponential equations. Where exponential equation for the first part of the time course could not been fitted due to the large signal loss in the dead time, the K_d_ was estimated by plotting the amplitudes of the fluorescence decrease against ligand concentration followed by fitting a hyperbole.

### Limited trypsinolysis

The limited tryptic digestion of dUTPases was performed at 37 ^o^C using 0.5 mg/ml protein concentration and 1:20 trypsin: dUTPase ratio in assay buffer also containing either 0.1 mM EDTA or 5 mM MgCl_2_. The tryptic digestion was terminated by the addition of 1 mM PMSF to the samples taken at different time points. The time dependence of the trypsinolysis was analyzed on SDS-PAGE. Limited trypsinolysis in the presence of 1 mM α,β-imido-dUTP was performed likewise in the same buffer either with or without Mg^2+^. SDS-PAGE gels were analyzed by densitometry with the UVIdoc software.

The cleavage of the linkers of the WWN covalent enzymes was performed at 25 °C using 0.8 mg/ml protein concentration and 1:500 trypsin: dUTPase ratio in the assay buffer also containing 1 mM α,β-imido-dUTP for the protection of the C-terminus^32^ and 5 mM MgCl_2_. The digestion was terminated by the addition of 1 mM benzamidine hydrochloride after 5 minutes. The sample was dialyzed against assay buffer containing 1mM benzamidine and 5 mM MgCl_2_ to remove α,β-imido-dUTP. The control sample was treated similarly without the addition of trypsin. The trypsinolysis product, the W/W/N heterotrimer, was analysed on SDS-PAGE and by enzymatic assays.

### Data fitting and statistical analysis

Data fitting was performed using Origin 7.5 (OriginLab Corp., Northampton, MA) or the stopped-flow software. Error bars represent the standard deviation of the mean of several measurements depending on the type of assay (detailed at each Method section and in the legends of the Figures and Tables).

## Additional Information

**How to cite this article**: Szabó, J. E. *et al.* Trading in cooperativity for specificity to maintain uracil-free DNA. *Sci. Rep.* **6**, 24219; doi: 10.1038/srep24219 (2016).

## Supplementary Material

Supplementary Information

## Figures and Tables

**Table 1:** Kinetic parameters.

	k_cat_ (s^−1^)^##^	k_sto_(s^−1^)^##^	K_M_ (μM)^#^	K_d,dUTP_ (μM)^#^
hDUT^F158W^	8 ± 3*	6.4*	3.6 ± 1.9*	0.83*
WWW	6.9 ± 0.5	6.33 ± 0.03	1.7 ± 0.5	0.29 ± 0.02
WWF	4.1 ± 0.5	6.13 ± 0.05	0.4 ± 0.3	0.29 ± 0.10
WWN	3.7 ± 0.3	6.31 ± 0.05	0.4 ± 0.3	0.59 ± 0.08
WNN	2.0 ± 0.1	5.72 ± 0.13	2.1 ± 0.5	0.29 ± 0.03
WWS	3.2 ± 0.3	6.26 ± 0.04	2.5 ± 1.2	0.70 ± 0.02

*Data from Toth *et al.* JBC^24^.

^#^Errors represent the fitting error.

^##^Errors represent the standard error (SE).
