# Supplementary material for: Trading in cooperativity for specificity to maintain uracil-free DNA
Source: Sci Rep. 2016 Apr 11;6:24219. doi: 10.1038/srep24219 (PMC4827122; doi:10.1038/srep24219)
Supplement: Supplementary Information [file srep24219-s1.pdf]

Trading in cooperativity for specificity to maintain uracil-free DNA

Judit E. Szabó[1], Enikő Takács[1], Gábor Merényi[1#], Beáta G. Vértessy[1,2], Judit Tóth[1 *]

[1]Institute of Enzymology, Research Centre for Natural sciences, Hungarian Academy of sciences, Budapest, Hungary
[2]Department of Applied Biotechnology and Food Science, Budapest University of Technology and Economics, Budapest, Hungary

* To whom correspondence should be addressed. Tel: +36-1-3826-707; Email: toth.judit@ttk.mta.hu

Present Address: Gábor Merényi, Department of Molecular Biosciences, The Wenner-Gren Institute, Stockholm University

Supplementary Results

The linkers in WWW do not influence the dUTPase enzymatic cycle

The WWW construct showed similar enzymatic properties to those of $hDUT^{F158W}$ under steady state conditions (V_{max} = 6.9 ± 1.2 s^{-1}, K_M = 1.3 ± 0.34 μM, Figure 4, Table 1). To confirm that the covalent linkage of the dUTPase monomers does not alter the enzymatic mechanism, we performed transient kinetic analysis as well. We determined the single turnover catalytic rate constant of the WWW enzyme (k_{STO} = 6.2 ± 0.2 s^{-}) and found it almost identical to the one reported for $hDUT^{F158W}$ (k_{STO} = 6.4 ± 0.2 s^{-1} (1)) (Supplementary Figure 1A, Table 1). The substrate binding properties of WWW also proved to be similar to those of $hDUT^{F158W}$ (Supplementary Figure 1B-C). WWW also binds dUTP in two steps. However, the observed rate constants for the collision complex formation were found to be higher than in the case of $hDUT^{F158W}$ indicating that that dUTP binds somewhat faster to the covalently linked enzyme. The observed rate constant of the second binding phase (proposed to be the isomerization of the enzyme-substrate complex (1)) did not depend on the dUTP concentration in the investigated concentration range (high above the K_M). In this concentration range, the observed rate constants for an isomerization step approximate the sum of the forward and the backward rates of the suspected conformational change. In the kinetic model for the $hDUT^{F158W}$, the rate constants for the isomerization step are k_{iso}= 21.2 s^{-1} and k_{-iso}= 3.7 s^{-1} (1). Therefore, the obtained k_{obs2} = 30.4 ± 9.13 $s^{-1}$for dUTP binding to WWW corresponds well to the isomerization observed rate constants of a wild type enzyme. This indicates that this step of the enzyme reaction remains unaltered by the linkage of dUTPase monomers. The concentration dependence of the total fluorescence intensity change of the stopped flow time courses was used to determine the K_d of the enzyme.dUTP complexes (Figure 4D). All these parameters determined for WWW and previously for $hDUT^{F158W}$ can be compared in Table 1.

In summary, the only notable change in the enzymatic mechanism was detected in the kinetics of the collision complex formation with the substrate. We propose that the increase in the rate constant of the first dUTP binding step may be due to the altered flexibility of the swapping arm due to the inserted peptide linker. The swapping arm confers the P-loop-like motif that participates in dUTP binding and hydrolysis (2).

Characterization of the mutations applied to create the asymmetric hybrid enzymes

We designed a mutation to impair dUTP binding to the active site of dUTPase. We introduced an aromatic ring into the tight ß hairpin of the uracil binding pocket of the active site by the replacement of Ala98 with the bulky Phe. The Phe was expected to prevent ligand binding by excluding its uracil moiety from its cognate binding site (Supplementary Figure 3B). To characterize the mutation, we first created a homotrimer containing the A98F change in each of the three active sites. As expected, the $hDUT^{F158W,\ A98F}$ homotrimer did not exhibit any dUTPase activity (Supplementary Figure 3A). To exclude that the lack of enzymatic activity resulted from a compromised protein structure, we

measured the thermal unfolding properties of the $hDUT^{F158W, A98F}$ protein. The thermal unfolding curve of $hDUT^{F158W, A98F}$ was cooperative and yielded identical melting temperature (T_m) to that of $hDUT^{F158W}$ (Supplementary Figure 3C-D). As the binding of a cognate ligand to the enzyme stabilizes its structure and thus shifts the T_m of the complex higher, we performed the experiment in the presence of saturating deoxyuracil nucleotides as well (dUMP and dUTP). While a large increase in T_m was observed in the wild type enzyme-nucleotide complexes, the unfolding curve of the $hDUT^{F158W, A98F}$ protein remained unaffected by the nucleotides (Supplementary Figure 3C-D). To further test the stability and nucleotide binding ability of the $hDUT^{F158W, A98F}$ protein, we performed limited trypsinolysis experiments in the presence and absence of nucleotides as well. It was shown earlier that the flexible N- and C-termini become readily cleaved in the apo enzyme while the substrate analog dUPNPP protects the C-terminus against tryptic cleavage (3). The resulting protein core remains stable for long time(3). In case of the apo enzyme, the limited trypsinolysis resulted in similar digestion patterns in both $hDUT^{F158W}$ and $hDUT^{F158W, A98F}$ (Supplementary Figure 3E). The protection effect of dUPNPP was well observable in $hDUT^{F158W}$ but was absent in $hDUT^{F158W, A98F}$ (Supplementary Figure 3E). Both the thermal unfolding and the limited trypsinolysis results confirm that the $hDUT^{F158W, A98F}$ protein is well folded but is not able to bind dUTP or dUMP.

The effect of Mg^{2+} binding to WWW

To reinforce the effect of Mg^{2+} binding to the central channel on the global structure of dUTPase, we repeated the CD experiments carried out with $hDUT^{F158W}$ (Figure 5B-C) using the WWW construct as well. The near-UV CD spectrum of WWW (Supplementary Figure 5A) showed some differences in the Phe region (250-270 nm) compared with that of $hDUT^{F158W}$. The $hDUT^{F158W}$ protein contains a Phe (F48) in the flexible N-terminal part. The presence of the linker in WWW probably changes the conformational properties of the linked N-terminus in the second and third subunits. The N-terminal part of the human dUTPase is involved in nuclear import (4) but not in the catalytic reaction (3, 4). The addition of $MgCl_2$ resulted in the same characteristic spectral changes as in $hDUT^{F158W}$. The far-UV CD spectrum showed no changes upon the addition of $MgCl_2$, similarly to what was observed in $hDUT^{F158W}$ (Supplementary Figure 5B).

Altogether, these results reinforce that Mg^{2+} binds to the unliganded dUTPase structure and evokes a stabilization effect.

Supplementary References

1. Tóth,J., Varga,B., Kovács,M., Málnási-Csizmadia,A. and Vértessy,B.G. (2007) Kinetic mechanism of human dUTPase, an essential nucleotide pyrophosphatase enzyme. *J. Biol. Chem.*, **282**, 33572–82.

2. Pécsi,I., Szabó,J.E., Adams,S.D., Simon,I., Sellers,J.R., Vértessy,B.G. and Tóth,J. (2011) Nucleotide pyrophosphatase employs a P-loop-like motif to enhance catalytic power and NDP/NTP discrimination. *Proc. Natl. Acad. Sci. U. S. A.*, **108**, 14437–42.

3. Varga,B., Barabás,O., Kovári,J., Tóth,J., Hunyadi-Gulyás,E., Klement,E., Medzihradszky,K.F., Tölgyesi,F., Fidy,J. and Vértessy,B.G. (2007) Active site closure facilitates juxtaposition of reactant atoms for initiation of catalysis by human dUTPase. *FEBS Lett.*, **581**, 4783–8.

4. Bozóky,Z., Róna,G., Klement,É., Medzihradszky,K.F., Merényi,G., Vértessy,B.G. and Friedrich,P. (2011) Calpain-catalyzed proteolysis of human dutpase specifically removes the nuclear localization signal peptide. *PLoS One*, **6**, e19546.

5. Kovári,J., Barabás,O., Takács,E., Békési,A., Dubrovay,Z., Pongrácz,V., Zagyva,I., Imre,T., Szabó,P. and Vértessy,B.G. (2004) Altered active site flexibility and a structural metal-binding site in eukaryotic dUTPase: kinetic characterization, folding, and crystallographic studies of the homotrimeric Drosophila enzyme. *J. Biol. Chem.*, **279**, 17932–44.

6. Barabás,O., Pongrácz,V., Kovári,J., Wilmanns,M. and Vértessy,B.G. (2004) Structural insights into the catalytic mechanism of phosphate ester hydrolysis by dUTPase. *J. Biol. Chem.*, **279**, 42907–15.

Supplementary Table 1

Name	For/Rev	R. site	Sequence	Goal
**D102N**	for	-	GGAGCTGGTGTCATAAATGAAGATTATAGAGGAAATGTTGG	Mutagenesis
	rev	-	CCAACATTTCCTCTATAATCTTCATTTATGACACCAGCTCC	
**A98F**	for	-	GATGTAGGATTTGGTGTCATAGATG	Mutagenesis
	rev	-	CATCTATGACACCAAATCCTACATC	
**Subunit I.**	for	Kpn1	GGGGTACCATGCCCTGCTCTGAAGAG	Assembly of WWW with linkers
	rev	BamH1	CGGGATCCGGTCGCGCCGCTGGTGCCGCCTTCGCTGCCGCCGCCTTCGCTGCCGCCCGCGCCGCTCGCATTCTTTCCAGTGGAACC	
**Subunit II.**	for	BamH1	CGGGATCCATGCCCTGCTCTGAAGAG	
	rev	Pst1	AACTGCAGGGTCGCGCCGCTGGTGCCGCCTTCGCTGCCGCCGCCTTCGCTGCCGCCCGCGCCGCTCGCATTCTTTCCAGTGGAACC	
**Subunit III.**	for	Pst1	AACTGCAGATGCCCTGCTCTGAAGAG	
	rev	Hind3	CCCAAGCTTTTAATTCTTTCCAGTGGAACC	
**Subunit III. change**	for	Pst1	CTACGCGGCTGCAGATGCCCTGCTCTGAAGAGACAC	Subunit III. exchange
	rev	Xho1	GCGCCAGCTCGAGTTAATTCTTTCCAGTGGAACC	
**Linker1:**			ASGAGGSEGGGSEGGTSGATGS	
**Linker2:**			ASGAGGSEGGGSEGGTSGATLQ	

Supplementary Figures

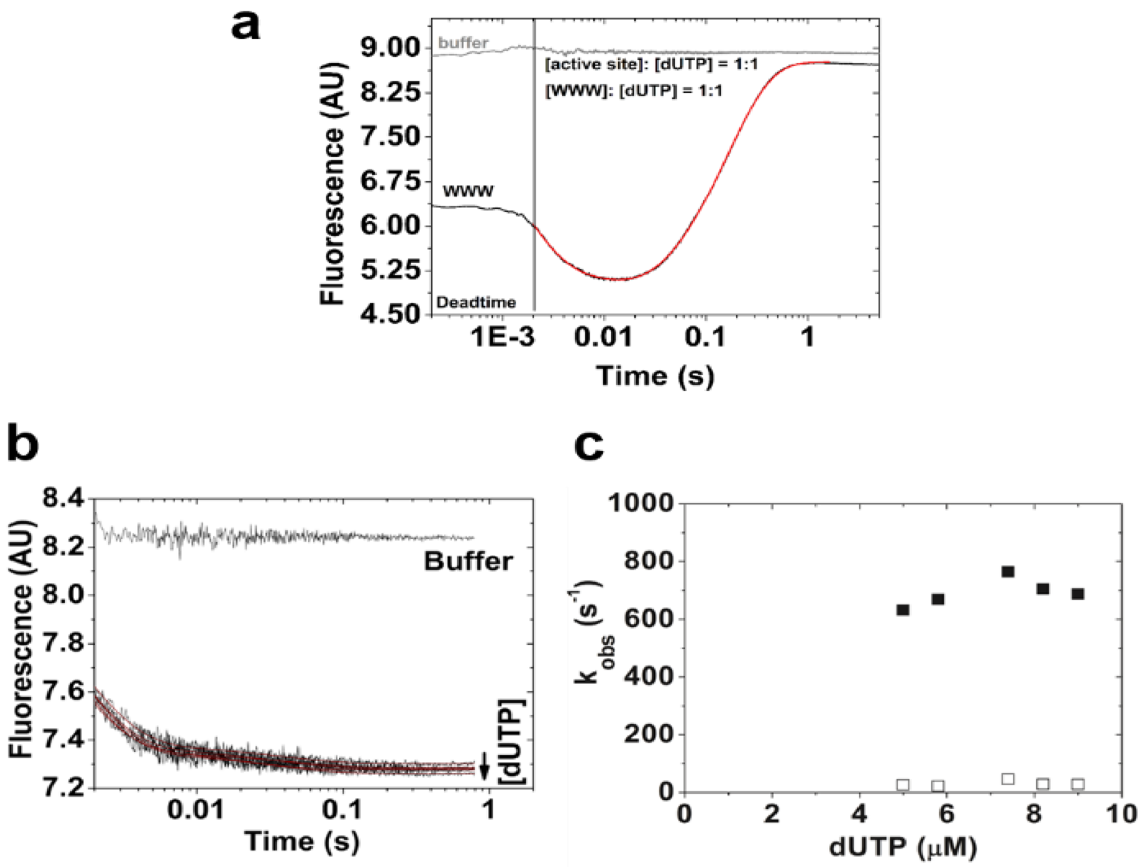

Supplementary Figure 1

Transient kinetic analysis of dUTP binding and hydrolysis by the WWW enzyme

A, Fluorescence time courses recorded upon mixing 20 μM WWW with substoichiometric dUTP or with buffer. The single turnover trace was fitted with triple exponential function (red curve) that yielded 6.24 s^{-1} for the single turnover rate constant (k_{STO}). Both the value of the k_{STO} and the characteristic fluorescence changes of the time course denote wild type enzymatic behavior(1). ***B,*** Fluorescence time courses recorded upon mixing various concentrations of dUTP with 0.5 μM WWW (postmixing concentration). Smooth lines are double exponential fits to the curves. A large fraction of the amplitude is lost in the dead time of the stopped flow instrument. ***C***, Analysis of the rate constants of the dUTP binding time courses in panel B. The solid squares denote $k_{obs,1}$ for the fast phase of the fitted double exponential. Since a large portion of the amplitude is missing, the concentration dependence of $k_{obs,1}$ did not yield exact association and dissociation constants. Open squares denote $k_{obs,2}$ of the second phase. $k_{obs,2}$ is independent of the dUTP concentration and its mean value is 30.4 ± 9.13 s^{-1}. Based on these results, the WWW enzyme is indistinguishable from the hDUT^{F158W} wild type enzyme (1).

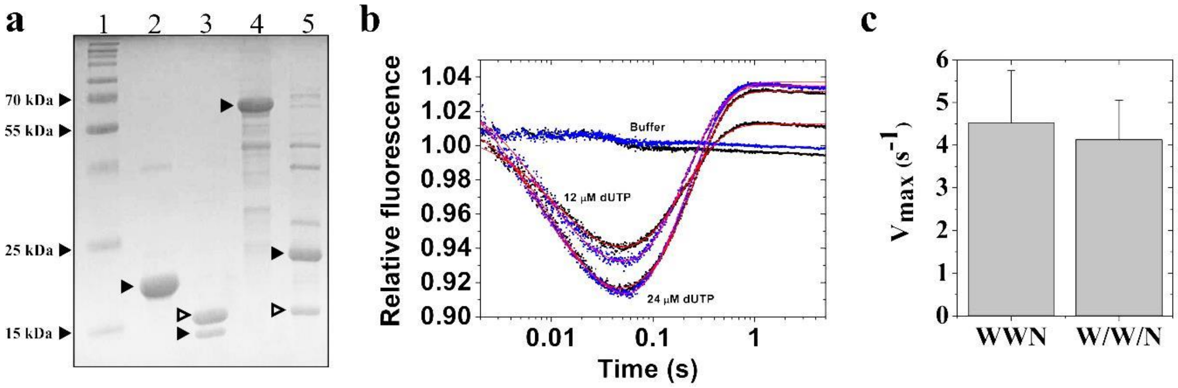

Supplementary Figure 2

Covalent coupling of the dUTPase monomers does not disturb the enzymatic properties of the functional trimers

A, PAGE analysis of the products of limited trypsinolysis. Lane 1: marker; lane 2: hDUT; Lane 3: hDUT treated with tryspin (empty arrow head: N-terminal cleaved protein fragment, black arrowhead: N- and C-terminal cleaved protein fragment); Lane 4: WWN covalent heterotrimer; Lane 5: WWN heterotrimer treated with tryspin. dUPNPP was present to give protection against the cleavage of the C-terminus (contains the catalytically important conserved motif V). The black arrowhead and the empty arrowhead points to monomers with linkers (2 monomers / trimer) and without linker (one monomer / trimer), respectively. Note that an N- and C-terminally cleaved fragment is not present thanks to the protective effect of the bound dUPNPP. ***B,*** Single turnover analysis of intact (WWN) and cleaved (W/W/N) heterotrimers measured by stopped flow. k_{STO} values derived from triple exponential fits (red curves) to the time courses were 5.5 ± 0.1 s^{-1} (12 μM dUTP) and 5.9 ± 0.1 s^{-1} (24 μM dUTP) for WWN; 5.6 ± 0.1 s^{-1} (12 μM dUTP) and 5.3 ± 0.1 s^{-1} (24 μM dUTP) for W/W/N. Errors represent fitting errors. ***C,*** Steady state activity of the WWN (4.5 ± 1.2 s^{-1}, n = 3) and of the W/W/N (4.1 ± 0.9 s^{-1}, n = 2) enzymes.

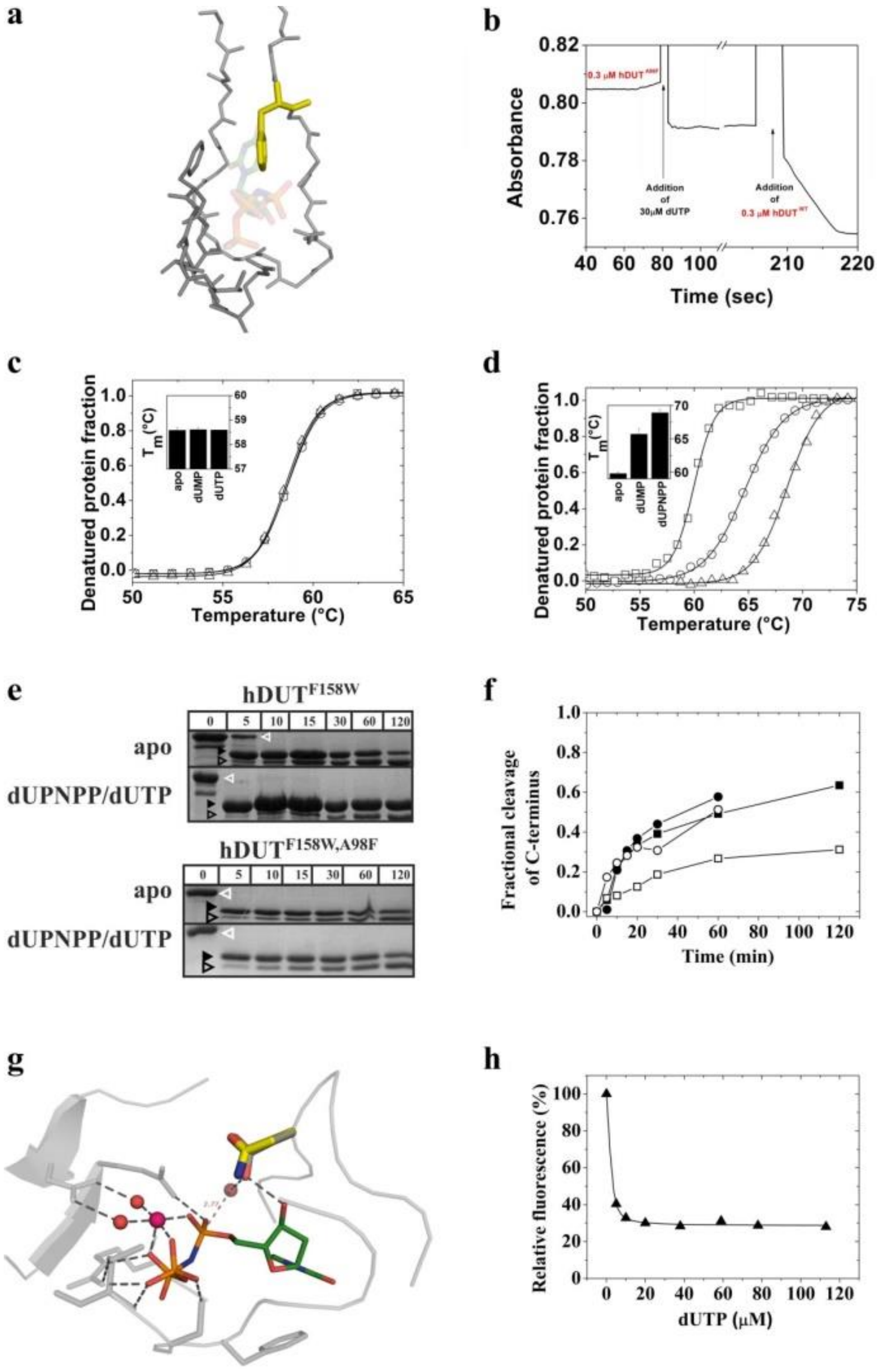
a
b
0.82
0.80
0.78
0.76
Absorbance
0.3 μM hDUT^A98F
Addition of 30μM dUTP
Addition of 0.3 μM hDUT^WT
40 60 80 100 210 220
Time (sec)
c
Denatured protein fraction
1.0 0.8 0.6 0.4 0.2 0.0
T_m(°C)
60 59 58 57
apo dUMP dUTP
50 55 60 65
Temperature (°C)
d
Denatured protein fraction
1.0 0.8 0.6 0.4 0.2 0.0
T_m(°C)
70 65 60
apo dUMP dUPNPP
50 55 60 65 70 75
Temperature (°C)
e
hDUT^F158W
0 5 10 15 30 60 120
apo
dUPNPP/dUTP
hDUT^F158W,A98F
0 5 10 15 30 60 120
apo
dUPNPP/dUTP
f
Fractional cleavage of C-terminus
1.0 0.8 0.6 0.4 0.2 0.0
0 20 40 60 80 100 120
Time (min)
g
h
Relative fluorescence (%)
100 80 60 40 20 0
0 20 40 60 80 100 120
dUTP (μM)

Supplementary Figure 3

Characterization of the A98F and the D102 mutations

A, Structural model of the active site of the A98F mutant hDUT (PyMol). The uracil-binding ß hairpin and the 5th motif from the swapping arm of human dUTPase are shown as grey stick backbone. dUTP is shown as transparent sticks with atomic coloring. The single allowed conformation of Phe98 in this structure is highlighted by yellow coloring. This structural representation shows that the aromatic ring of Phe98 occupies the binding site of the uracil moiety of the substrate. ***B***, Enzyme activity assay. The A98F protein is premixed in the assay buffer, the dUTPase reaction is initiated by the addition of dUTP. No enzyme activity is detected until the addition of the wild type enzyme that hydrolyzes the intact dUTP. ***C-D***, Thermal unfolding of the A98F mutant (***C***) and of the wild type dUTPase (***D***), respectively, in the absence of nucleotides (square), in the presence of 5 mM dUMP (circle) or 1 mM dUTP (triangle). Smooth lines through the data are Boltzmann fits (Equation (4))yielding the melting temperatures presented as bar graph in the inset. ***E***, Limited trypsinolysis of the wild type (hDUT^{F158W}) and the A98F mutant (hDUTF$^{158W,\ A98F}$) in the absence and in the presence of 1 mM dUPNPP. Numbers denote the duration of the tryptic treatment in minutes. The open white arrow head, the solid black arrow head and the open black arrow head shows the intact, the N-terminally cleaved and the N- and C-terminally cleaved enzyme, respectively. ***F,*** Densitometric analysis of the limited trypsinolysis experiment. The graph shows the relative amount of the N- and C-terminally cleaved protein compared to the total amount of protein. It has previously been established that trypsin readily cleaves the flexible N-terminus of dUTPases in a nucleotide-independent manner, while the binding of dUTP or dUPNPP to the enzyme protects the also flexible C-terminus from tryptic digestion (5). We observe this protection in the wild type enzyme but not in the A98F mutant (see apo vs. dUPNPP/dUTP samples on the gel at 120 min). These data together demonstrate that the A98F substitution hinders the binding of dUTP to the active site while it does not perturb the overall structure (folding, stability) of the protein. ***G***, Structural representation of the D102N mutation in the active site of hDUT (generated by Pymol). The active site building amino acids are shown as grey cartoon and stick backbone. dUTP and residue 102 are shown as sticks with atomic coloring. The Mg^{2+} ion is shown as a magenta sphere, the water molecules are shown as red spheres. D102 coordinates the nucleophile catalytic water molecule while the mutant D102N is not expected to efficiently coordinate this water molecule (6). ***H,*** Fluorescence intensity titration is shown upon dUTP binding to hDUT$^{F158W,\ D102N}$. The smooth line through the data is a quadratic fit (Equation (1)) yielding K_d = 0.7 μM. The quasi wild type relative fluorescence change and K_d (cf. data from the literature (1)) indicate that the substrate binding properties of the hDUT$^{F158W,\ D102N}$ mutant remained unaffected by the D102N mutation.

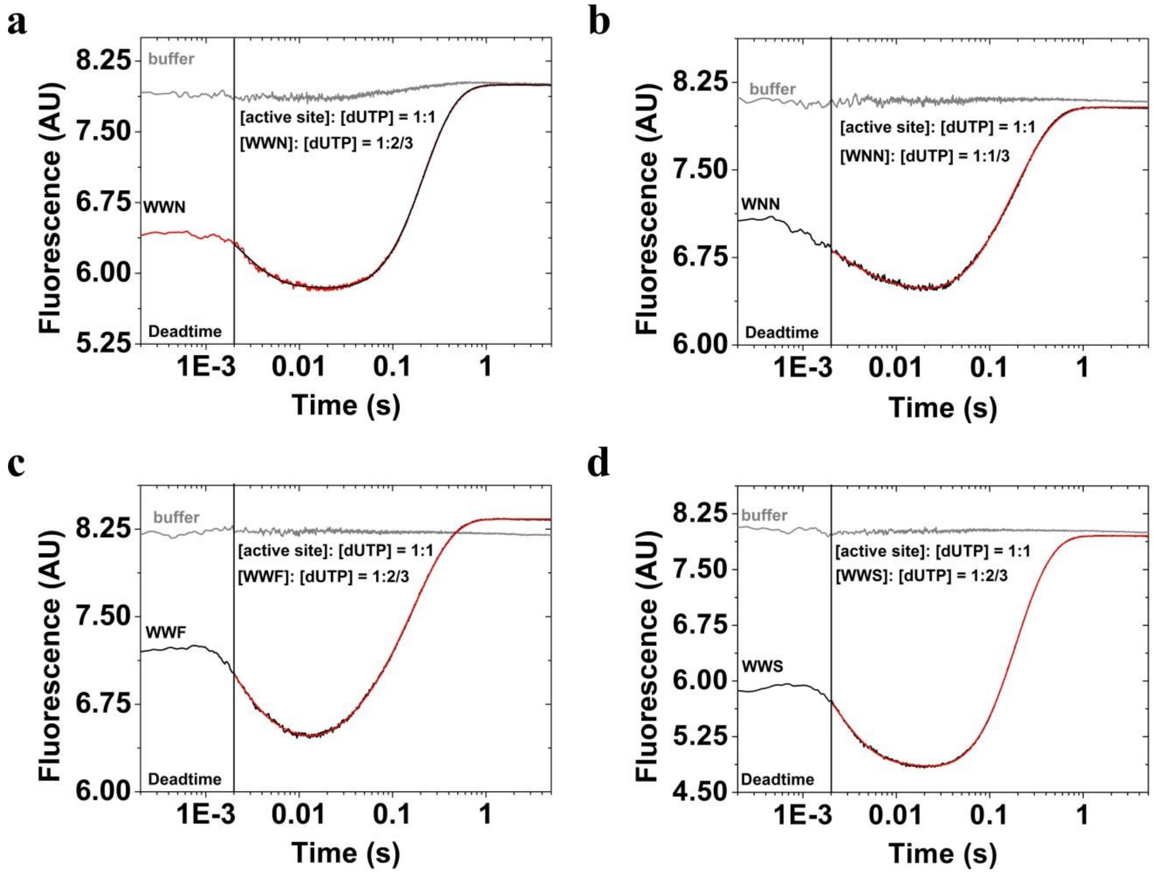

Supplementary Figure 4

Single turnover dUTP hydrolysis time courses of the hybrid enzymes

Fluorescence time courses were recorded upon mixing substoichiometric dUTP with 20µM WWN (***A***), WNN (***B***), WWF (***C***) or WWS (***D***). Single turnover traces were fitted with triple exponential function yielding the following k_{STO} values: 6.43 s^{-1} for WWN, 5.4 s^{-1} for WNN, 5.99 s^{-1} for WWF and 6.26 s^{-1} for WWS. Each measurement was repeated several times, the mean values and errors are shown in Figure 4C and in Table 1. Note that the signal changes are not comparable dues to the optimization of the detector sensitivity in each measurement.

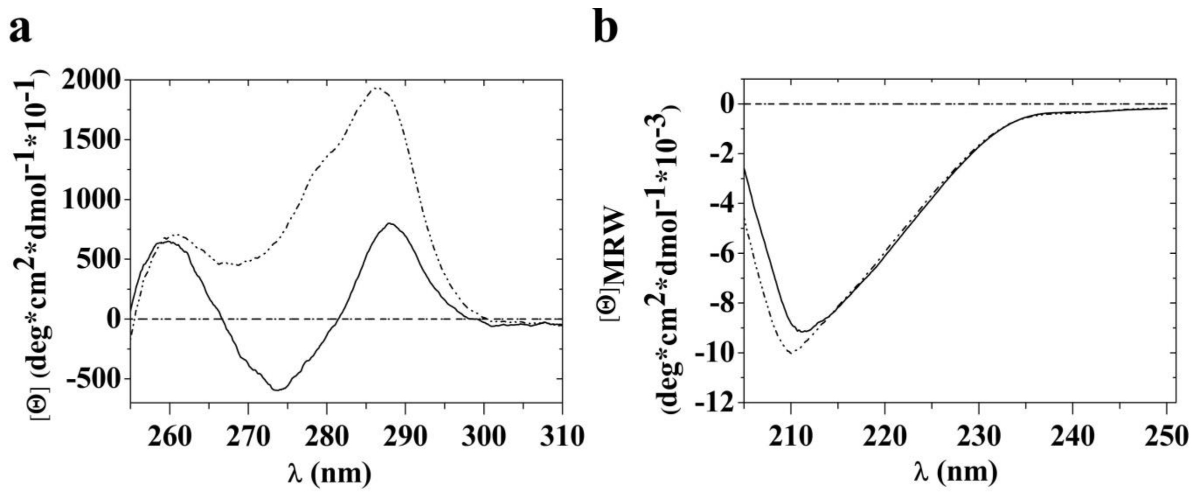

Supplementary Figure 5

The Mg^{2+} binding properties of WWW are similar to those of $hDUT^{F158W}$ (cf. Figure 5)

A, Near UV and ***B,*** far UV CD spectra of WWW in the presence (dash-dot-dot) and in the absence (solid line) of Mg^{2+}. The buffer signal is shown as dash-dash-dot lines.

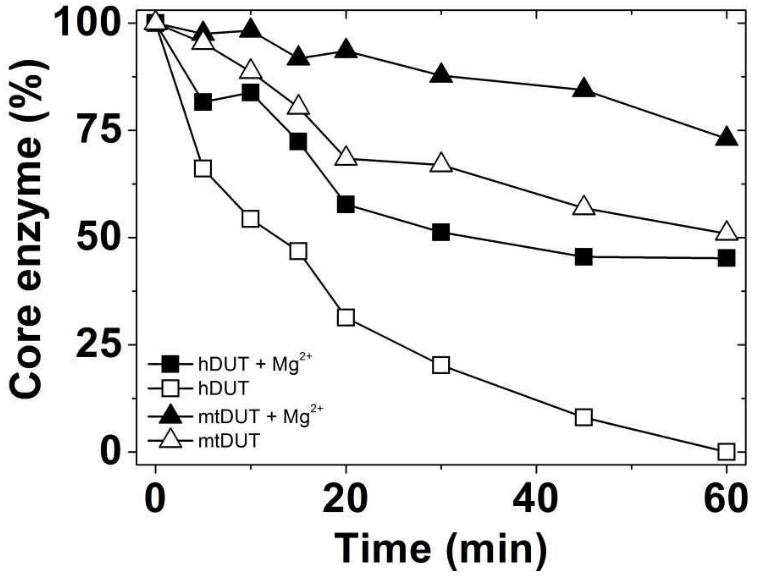

Supplementary Figure 6

Densitometric analysis of the limited trypsinolysis experiment shown in Figure 5D

The relative amount of core enzyme is plotted against time. Core enzyme = intact enzyme + N-terminal cleaved enzyme + N- and C-terminal cleaved enzyme
